# Treatment needs, diagnoses and use of services for acutely admitted psychiatric patients in northwest Russia and northern Norway

**DOI:** 10.1186/1752-4458-7-4

**Published:** 2013-01-14

**Authors:** Knut W Sørgaard, Grigory Rezvy, Anatoly Bugdanov, Tore Sørlie, Trond Bratlid

**Affiliations:** 1 Nordland Hospital Trust, Bodø, 8092, Norway; 2 Department of Clinical Medicine, University of Tromsø, Tromsø, 9001, Norway; 3 North State Medical University, Arkhangelsk, Russia; 4 Arkhangelsk Clinical Psychiatric Hospital, Arkhangelsk, Russia

**Keywords:** Russian psychiatry, Acute psychiatry, Inpatient treatment, Comparative studies

## Abstract

**Background:**

We compared demography, diagnoses and clinical needs in acutely admitted psychiatric hospital patients in northwest Russia and northern Norway.

**Method:**

All acutely admitted psychiatric patients in 1 psychiatric hospital in north-west Russia and 2 in northern Norway were in a three months period assessed with HoNOS and a Norwegian form developed to study acute psychiatric services (MAP). Data from a total of 841 patients were analysed (377 Norwegian, 464 Russian) with univariate and multivariate statistics.

**Results:**

Russian patients were more often males who had paid work. 2/3 were diagnosed with alcohol and organic disorders, and 70% reported problems related to sleep. Depression was widespread, as were problems associated with occupation. Many more Norwegian patients were on various forms of social security and lived in community supported homes. They had a clinical profile of affective disorders, use of drugs, suicidality and problems with activities involved of daily life. Slightly more Norwegian patients were involuntary admitted.

**Conclusion:**

Acutely admitted psychiatric patients in North West Russia and Northern Norwegian showed different clinical profiles: alcohol, depression and organic disorders characterised Russian patients, affective disorders, suicidality and use of drugs characterised the Norwegians. Whereas Norwegian patients are mainly referred from GPs the Russians come via 1.line psychiatric services (“dispensaries”). Average length of stay for Russian patients was 2.5 times longer than that of the Norwegian.

## Background

Cultural, political and economic values strongly influence how human services systems are organized and how they operate. In this paper we compare acutely admitted psychiatric patients in north-west Russia and in northern Norway with regard to demography, admission characteristics and treatment needs. Northern Norway and north-west Russia are sub-arctic and mainly rural areas with some scattered urban or semi-urban centres. Differences exist with regards to demography, history, culture and economy. Whereas Norway has profited from decades of political stability, is among the most affluent countries in the world and exemplifies the Scandinavian well fare model, Russia has suffered from decades of authoritarian political regimes, and later - as other East European countries – has endured considerable social changes that has led to what has been called a “community syndrome”: increasing death rates, more depression, addiction, cerebrovascular and cardiovascular problems, and destructive and self destructive behaviour [1-5]. It is documented that social deprivation, lack of stable housing and community based services contribute to increased use of acute psychiatric services [6,7]. In Russia [6], as in Western Europe [7,8], the acute ward may come under pressure due to hospital downsizing, a rising number of admissions, staffing problems and patients with complex needs and challenging behaviour [7,9]. There have been few international comparative studies on acute psychiatric services [10] and Eastern Europe is seldom included [11]. The WHO 17-countries study of use of mental health services concluded that the effects of different mental health policies, delivery systems, and financing “is essentially unknown” and that detailed data relevant to these topics should be collected [12]. Russian psychiatry is not well known in Western Europe. Psychiatric care in Northern Norway and the Arkhangelsk region has previously been systematically compared at a treatment system level [13] by our group. The present study is intended to supplement this by adding clinical and demographic data of the users of acute psychiatric services in north west Russia (the Arkhangelsk region) and northern Norway.

We expected to find: (a) Alcohol/drug and organic problems would be more common among Russian patients, whereas among the Norwegian patients affective problems and problems related to suicidality would dominate. (b) Due to more deprived social and economic conditions, serious mental problems (as measured by HoNOS) would be more common among the Russian patients. (c) A less developed social security system in Russian would cause Russian patients to stay in ordinary employment whereas more Norwegian patients would live on social security.

### The context of the study

Confinement, a strong belief in science and close contacts with the political system are characteristics of psychiatry in the Soviet period [14]. Since the beginning of the 1990s, there has been a gradual acceptance of the bio-psycho-social model, diagnostic and clinical guidelines more in accordance with European standards, reduction in the number of beds, and multiprofessional teambuilding. The collapse of the economy in the 1990s reduced governmental financing and caused difficulties for patients and professionals [14]. New psychotropics are available, but their uses depend on the region’s funding [15]. There is no national health insurance comparable to those in Western Europe [15]. Russia established a national law on psychiatry in 1992 [14] that is comparable to the Norwegian one [13] and the services have gradually developed in the direction of European standards: decentralising, strengthening of social psychiatric approaches, incorporating new treatment methods, and integrating psychiatry and somatic medicine. The primary care and the social services are still peripheral in the treatment of people with mental disorders [16]. Bed capacities are to a large extent centralised to hospitals with more than 1000 beds, and in 2006 the average length of stay for all patients was 77.4 days [17]. Outpatient services, mainly “dispensaries” staffed with psychiatrists are well developed in urban [13] and psychiatric “psychotherapy-cabinets”, are established in rural areas [17]. Health care development is increasingly based on epidemiological studies [13]. Both Arkhangelsk County and Northern Norway are mainly rural areas with a low population density, particularly Arkhangelsk with its 1.3 mil inhabitants living in an area covering 587 000 square kilometres. About 400, 000 lives in the city of Arkhangelsk and ca. 200, 000 in Severodvinsk. In Northern Norway 470, 000 inhabitants live in an area of 113 000 square kilometres – one third Norway’s territory. The number of emergency beds per 100 000 inhabitants in Arkhangelsk is about the same as in northern Norway [13]. The Arkhangelsk psychiatric hospital included in the present study, has about 900 beds, nine acute wards with 50-70 patients each and low staffing, approximately about 2-4 nurses and 4 nurse auxiliaries at each shift. In the last ten years, there has been systematic staff training in milieu therapy and multiprofessional cooperation in selected units [18,19]. A system of crises services has been established [17]. In Northern Norway, the principles of ‘regionalisation’ and ‘sectorisation’ predated a network of 14 community mental health centres (DPS – District Psychiatric Centres) that together with 2 downsized mental hospitals are the main components of the mental health system. There are 247 beds in the two hospitals, 69 of them in acute wards. The number of beds in a typical acute ward is 10-12, the staff consists of about 25 nurses/nurse auxiliaries in addition to psychologists (1-2), psychiatrists (1-2) and social workers (1). Each shift (daytime) may consist of 6-8 nurses/nurse auxillaries in addition to available psychologist, psychiatrist and social worker in wards with 10-14 beds. The standard procedure is short hospital stays and a rapid return to the patients’ homes in close collaboration with the primary health services in the patients’ home municipalities. Patients in need of specialized psychiatric follow-up are referred to the DPSs. All DPSs have mobile acute teams [20]. In addition, the municipalities operate a differentiated network of psychiatry-related services (GPs, social services, psychiatric nurses and psychiatric day care centres). The degree of decentralization in the mental health services is much higher in Northern Norway than in Russia [13] and the GPs have a more central role in the treatment of mental disorders.

## Methods

This study was an observation study with demographic data collected at admission, clinical information (symptoms, treatment-relevant information etc.) recorded at admission and at discharge. The data collection period was 3 months. A total of 983 admissions were included, but due to a number of readmissions (105 in Norway and 55 in Russia) that might result in clustered data, the analyses are based on the participants’ first admission in the study period. A total of 841 admissions were analysed (377 Norwegian, 464 Russia). The patients' therapists (psychiatrists or psychologists) were responsible for the data collection which took place in connection with ordinary clinical interviews. A form with 67 variables was filled out for each admission by the patients’ therapists in collaboration with other staff who knew the patient. The form was originally developed for use in the national Norwegian acute ward study – the MAP study [18] and has 8 sections and 67 variables: (A) referral and admission, (B) demographic data about the patient, (C) service received before the admission, (D) assessments made at admission, (E) systematic assessment and treatment made during the stay, (F) coordination and collaboration, (G) evaluation at discharge, and (I) data about the discharge. The HoNOS [19-21] is integrated in it. HoNOS is generally used for describing the pathology and clinical (8 items) and social needs (4 items) across 12 broad mental-health related dimensions [22-24]. It consists of 12 5-point scales from 0 (no problem) to 4 (severe/very severe problem). It was developed at the UK Royal College of psychiatrists as a routine outcome measure in mental health services. HoNOS was recorded at admission and at discharge.

In the present study, the forms and training material (case vignettes) were translated from Norwegian/English into Russian by one of the authors (GR), back translations were performed and necessary adjustments made. Therapists responsible for the HoNOS completed a one-week training course. The training started with a thorough presentation of the instruments, a number of case vignettes were rated, the scores were compared consecutively and discussed in the groups with the instructors present. To reduce ambiguity in the variable interpretation, written definitional criteria were available for the therapists. The HoNOS instructors could also easily be reached (emailing, telephone). The ethical committees in Northern Norway and at the Medical University in Arkangelsk approved the study, and also accepted that patients unable to give informed consent were included. The reason was that excluding patients would make the study unrepresentative for the total group of acute ward patients. ICD-10 diagnoses were used [25].

### Statistics

Frequency analyses, chi-square, T-tests on the demographic- and admission-related data, and standard binary logistic regression were used to characterise the main differences between patients in the two systems with country as the dependent variable (0 = Norway, 1 = Russia). Logistic regression was performed due to it’s potential for predicting which of two categories (e.g. Russian vs Norwegian hospitals) a person was likely to be admitted to. Potential explanatory variables were chosen with a significance value of .25 on univariate analyses as criteria for inclusion [26]. Variables that were not mulitvariately significant (p ≤ .05) on the Wald statistics in the first step of the analyses, were removed and subsequent analyses run without them [27]. Based on the p ≤ .001 criterion for Mahalanobis distance, which is used to identify particularly influential cases, outliers were removed. Depending on the choice of strategies, regression analyses may give some more or less related models. The final choice of model was made from the principle of parsimony [26] which emphasizes that a simple model is better than a more complex one. Initially, a demographic model was tested out and subsequently clinical variables were added. The forced entry procedure was used.

## Results

Russian patients (Tables 1 and 2) were older, more often males, fewer lived alone, and they more often lived in houses/flats. More Norwegians had institutional care (lived in community based care homes), and were on social securities. Russian patients were referred from dispensaries and medical emergency services, most Norwegians from GPs or medical emergency services. Slightly more Russians were voluntary admitted. Compulsory observation is a specific Norwegians alternative: patients can be involuntary admitted for observation for a maximum of 20 days. They cannot be medicated against their will, but be transferred to ordinary compulsory admission. The Russian patients were diagnosed with mainly alcohol/drug and organic disorders, whereas affective disorders, psychosis and “other disorders” were common among the Norwegians. HoNOS (Table 3) showed that high scores on Other mental or behavioural problems, Problems with relationships and Depressed moods characterised both groups. The Russian profile was problem drinking and drug-taking, problems with occupation and/activities, and with living conditions and hallucinations/delusions. The Norwegian was characterized by non-accidental self-injury, problems of activities of daily living, overactive/aggressive/disruptive behaviour and cognitive problems. The most common “Other mental problems” were (Norwegian patients): anxiety (1/3) and sleep disorders (1/4), and (Russians patients) sleep disorders (70%) (Chi square 144.1, p = .000). *Logistic regression* (Table 4)*.* Russian patients were more often living in flats/houses, together with parents, more often had work related income (compared to social security), were marginally older and more often of male sex. Percentage of of correct classification was 56.7; Hosmer/Lemeshow Chi sq. 23.04, p = .003; Nagelknerk R^2^ .14. Adding clinical variables, a more distinct set of variables with stronger statistical values characterised the Russian patients than the Norwegians: organic disorders, alcohol/drug related problems and source of income, older age, living in houses/flats (compared to community based care), more problems related to work and activities, and from depression. Affective disorders, suicidality at admission and problems related to activities of daily living characterised the Norwegian patients. Percentage of correct classification increased to 83.6. Hosmer/Lemeshow Chi sq. 13.08, p = .09; Nagelknerk R^2^ .84.

**Table 1:** Demographic characteristics of the Norwegian and Russian patients

**Variable**		**Norway**	**Russia**	**P**
Age		39.9 (s.d. 14.6)	44.1 (s.d.14.3)	p = .000; 95% CI: -6.20/2.50
Sex	Female	171 (48.4%)	188 (38.8%)	Chi sq 7.62, p = .006
Marital status	Married, cohab.	60 (17.1%)	136 (29.4%)	Chi sq 76.70, p = 000
	Living alone	271 (58.9%)	136 (27.4%)	Chi sq 184.85 p = .000
Children	Have children < 18yrs)	86 (24.3%)	92 (19.8%)	NS
	No of children	.48	.27	T-test 51.56 p = .001
Housing/dwelling	House/flat	221 (62.4%)	367 (79.1%)	Chi sq 59.42 p = .000
	Institution/care unit	56 (15.8%)	9 (1.9%)	
	Parents/others	43 (12.1%)	65 (13.3%)	
	Homeless	17 (3.5%)	17 (3.4%)	
	Other	33 (6.7%)	13 (2.5%)	
Income	Paid work	30 (8.5%)	115 (24.8%)	
	Disability pension	160 (45.2%)	142 (30.6%)	
	Other social security	93 (26.3%)	4 (.9%)	Chi sq 215.08, p = .000
	Old age pension	22 (6.2%)	59 (12.7%)	
	Other	46 (13.0%)	61 (13.1%)	

T-tests, chi square. N= 841.

**Table 2:** Formalities of referral and admission, diagnosis and HoNOS-ratings

	**Variable**	**Norway**	**Russia**	**P**
Previous psychiatric treatment	Yes	298 (83.4%)	336 (72.4%)	Pearson Chi square 14.02 p = .001
Referred from	Patient him/herself	9 (2.5%)	35 (7.5%)	Pearson Chi square 314.2, p= .000
	GP	99 (28.0%)	5 (1.1%)	
	Casualty clinic	142 (40.1%)	156 (33.6%)	
	Psychiatric outpat. units	12 (3.4%)	220 (47.4%)	
	Other psychiatric services	44 (12.4%)	3 (0.7%)	
	Other	48 (13.6%)	45 (9.7%%)	
Juridical basis for admission	Voluntary admissions	214 (60.5%)	304 (66.2%)	Pearson Chi square 111.02, p = .000
	Compulsory observation	68 (19.2%)	0 (0%)	
	Compulsory admissions	68 (19.2%)	157 (33.8%)	
	Other	6 (1.5%)	0 (0%)	
The patient wanted to be admitted		209 (59.4%)	332 (69.4%)	Pearson Chi square 17.4 p = .000
Length of stay (days)		11.1 (14.1)	26.7 (19.0)	F 93.52 p = .000
Diagnosis ICD 10	Organic	10 (2.8%)	78 (16.8%)	Chi sq 40.91, p = .000
	Alcohol/drugs	29 (8.2%)	217 (46.8%)	Chi sq 142.09, p = .000
	Affective disorders	97 (27.4%)	17 (3.7%)	Chi sq 94.33, p = .000
	Psychosis	120 (33.9%)	109 (23.5%)	Chi sq 10.79, p = .001
	Other diagnosis	98 (27.2%)	43 (9.3%)	Chi sq 49.94, p=.000
HoNOS	Total scores at admission	14.41 (5.87)	15.38 (5.02)	F 7.82, P < .001
“Improvement” (HoNOS)	Difference in vs out rating of total scores	5.50	8.20	F 39.3, p < .000
GAF	Gaf F admission	36.2 (12.6)	38.9 (12.2)	F 1.99, p = .003
	Gaf S admission	39.3 (12.3)	40.2 (12.7)	NS

Chi-square and T-tests.

**Table 3:** HoNOS ratings Russian and Norwegian patients

	**Russia**		**Norway**	
**HoNOS**	**Nil to minor**	**Mild to severe**	**Nil to minor**	**Mild to severe**
HoNOS 1 (Overactive, aggressive, disruptive)	322 (69.5%)	141 (30.5%)	231 (61.3%)	146 (39.7%)
HoNOS 2 (Non-accidental self-injury)	428 (92.7%)	35 (7.3%)	261 (69.8%)	115 (30.2%)
HoNOS 3 (Problem drinking, drug-taking)	213 (46.4%)	246 (53.6%)	271 (72.8%)	101 (27.2%)
HoNOS 4(Cognitive problems)	386 (83.7%)	75 (16.3%)	270 (74.0%)	119 (26.0%)
HoNOS 5 (Physical illness, disability)	348 (75.2%)	121 (24.8%)	288 (76.6%)	109 (24.4%)
HoNOS 6 (Hallucinations, delusions)	179 (38.7%)	304 (61.3%)	175 (47.8%)	191 (52.2%)
HoNOS 7 (Depressed moods)	251 (54.2%)	224 (45.8%)	180 (49.0%)	187 (51.0%)
HoNOS 8 (Other mental or behavioural problems)	139 (34.6%)	300 (65.4%)	90 (24.7%)	274 (75.3%)
HoNOS 9 (Problems with relationships)	180 (39.1%)	299 (60.9%)	138 (37.1%)	234 (62.9%)
HoNOS 10 (Problems with activities of daily living)	359 (77.7%)	103 (22.3% )	201 (54.9%)	215 (45.1%)
HoNOS 11 (Problems with living conditions)	294 (64.1%)	174 (35.9%)	147 (83.4%)	76 (16.6%)
HoNOS 12 (Problems with occupation/activities)	168 (36.8%)	291 (63.2%)	247 (68.8%)	112 (31.2%)

**Table 4:** Direct logistic regression analysis of patient nationality as a function of demographic variables

**Variables**	**B**	**Wald**	**Odds Ratio**	**95% C.I for Exp (B)**	
				**Lower**	**Upper**
Demographic variables					
Age (MAP)	.020	14.91	1.02	1.01	1.03
Sex (MAP)	- .52	11.81	.59	.44	.80
Income from work (MAP)	1.29	42.07	3.64	2.32	5.68
Living in own flat/house (vs by parents, institution etc) (MAP)	.68	16.46	1.98	1.43	2.75
Total model:	Correct classifications: 56.7%; Hosmer/Lemeshow Chi sq. 23.04, p = .003; Nagelknerk R^2^ .14				
With clinical variables added					
Income from work (MAP)	1.44	16.60	4.23	2.11	8.46
Living in own flat/house (MAP)	.76	7.44	2.15	1.24	3.72
Depression (HoNOS)	.28	6.64	1.33	1.07	1.64
Problems related to activities of daily life (HoNOS)	- .82	40.66	.44	.34	.56
Problems related to occupation and activities (HoNOS)	1.07	92.73	2.91	2.34	3.61
Organic disorders (ICD-10)	2.32	25.91	10.21	4.17	24.97
Alcohol/drugs (ICD-10)	2.47	52.39	11.81	6.05	23.05
Affective disorders (ICD-10)	−1.72	21.33	.18	.09	.37
Use of drugs	- 1.60	46.87	.20	.13	.32
Risk of Suicidality at admission (MAP)	- .58	37.19	.56	.46	.67
Total model:	Correct classifications: 87.6%; Hosmer/Lemeshow Chi sq. 11.03, p = .18; Nagelknerk R^2^ .71				

Norway = 0, Russia = 1. Only significant variables are shown.

## Discussion

### (i) Use of services

80 of the Russian patients were referred from dispenseries (psychiatric outpatient clinics/ psychiatric “psychotherapy-cabinets”) and medical emergency services, in Norway 70% came from GPs and medical emergencies. This reflects a structural difference in the mental health services between the two countries: Russia has a network of 1.line psychiatric specialist services where Norway – and most Western countries - uses GPs. The use of civil commitment in Norwegian psychiatry is among the highest in Europe [28], and more Norwegian patients than Russians were committed. Norwegian patients had also more often received psychiatric inpatient treatment in the last 12 months before admission, whereas the proportion that had used outpatient service was almost identical. The length of stay for the Russian patients was about 2½ times longer than for the Norwegians. Thus, the revolving door profile was more pronounced in the Norwegian system. In addition to a later entry into the “deinstitutionalization-age”, probable explanations for the longer stays and slower admission/readmission cycles in Russia may be their relative lack of outpatient services, e.g. community teams, interagency collaboration [16] and aftercare services in the peripheral areas [29]. Due to this, hospital psychiatrists often try to complete the treatment of the patients before the discharge. This assumption is strengthened by Russian patients more often being considered to be symptom free in the periods between admissions and that fewer were considered as suffering from “deterioration of an existing illness”. This may reflect cultural differences in how “worsening vs. improvement” is interpreted, or it may relate to real treatment gains: Russian therapists rated their patients’ average improvement (that is: HoNOS in vs HoNOS out) as higher than their Norwegian colleagues. However, the assumption of longer hospital stays leading to more clinical improvement is not well supported by previous research [30].

### (ii) Demography and clinical needs

High scores on other mental or behavioural problems, problems with relaionships and depressed moods (all HoNOS) characterised both Russian and Nowegian patients. Diagnostically (ICD-10), more Russians suffered from alchol and/or drug abuse and had organic disorders. In spite of the prevalence of depressed moods (54%), only 9 Russian patients were diagnosed with affective disorders. On the HoNOS, the specific Russian problem areas were problem drinking and drug-taking, problems with occupation and activities, with living conditions and hallucinations/delusions. Among the Norwegians, the most comon diagnoses were affective disorders, psychosis and “other diagnoses”. No-accidental self-injury, activities of daily living, overactive/aggressive/disruptive behaviour and cognitive problems dominated the HoNOS-scores. According to Rezvy et al, compared to Russianpsychiatrists, the diagnostic practice of their Norwegian colleagues may show a tendency to focus on the affective aspects of schizoaffective disorders and overestimate the degree of depression in moderate depressive cases [31]. Nevertheless, based on our data there appear to be mismatch between the HoNOS ratings of depressed moods among Russian patients and the infrequent use of ICD-10’s affective disorders. (iii) *The multivariate analyses* showed the Russian patients to be characterised by organic disorders, alcohol related problems, depression, problems related to activities of daily life, to work and activities. They were more often employed and lived in houses/flats (in contrast to community based care homes). Affective disorders, suicidality at admission, use of drugs and problems related to activities of daily living characterised the Norwegian patients. The problems of alcohol abuse in Russia are well known [32,33] and organic disorders are obvious consequences. High prevalence of depression associated with alcohol and general problems of life style is reported in other studies from Eastern Europe, including Russia [5,34,35]. In the present study, 40.4% of the Russian patients had serious alcohol problems (continuous use of alcohol, use disturbing other activities, spending much time trying to get it) compared to only 8.3% of the Norwegians. A national Norwegian 2003 census-day study found that only 10% of all psychiatric inpatients had alcohol or substance abuse diagnoses [36]. On the other hand, serious use of drugs was more frequent among the Norwegians patients (10.1% vs 1.1%). Sleep problems accompanies both chronic and acute abstinence and may contribute to further drinking problems among persons with alcohol problems [37]. 70% of the Russian patients reported sleep problems.

Nock et al [38] found mood disorders to be a common risk factor of suicidality in high-income countries, whereas impulse-control disorders – related to for example alcohol abuse - were more dominant in low-and middle income countries. Suicide rates in Russia are linked to high alcohol-consumption [39]. Affective problems and suicidality were prominent in Norwegian patients, but in spite of widespread problems with drug and alcohol abuse and HoNOS-rated depressed moods, suicidality at admission affected only about 7% of the Russian patients (compared to 1/3 of the Norwegians). When rated during the stay, 2.7% of the Russian patients and 15.2% of the Norwegians had moderate to high suicidal risk. The low frequency of suicide related problems among the Russian patients may lead on to ask about how these problems are conceptualised and assessed in Russian hospitals. Acutely admitted Russian patients with observable alcohol problems are often admitted directly into the “narcological departments” (wards specializing in the treatment of comorbid psychiatric and drug/alcohol disorders) of psychiatric hospitals, which may lead to an underdiagnosing of disorders related to affective problems.

Norwegian patients had an average HoNOS-total at admission of 14.41 points, Russians 15.38. The HoNOS-scores from both countries did not differ substantially from what has been found in other inpatient studies [40-46], but due to the structural and demographic differences between the two countries, the difference may seem unexpectedly small. A partly explanation may be different attitudes towards admitting mental problems: In a comparative study, Angermeyer et al [10] found that Russian respondents had a stronger tendency to consider mental disorders as self-inflicted. Corresponding guilt and shame may prevent disclosure of mental health problems. There may also have been downward adaption to poorer living conditions among Russian patients.

### Employment

Compared to the Norwegian sample, a greater proportion of the Russian patients were employed (25% vs 8%), and far more Norwegian patients (71.5% vs 31.5%) were on social security. When we compared the score on the HoNOS-item that measures problems with occupation and daily activities, the difference between those who were employed and those who were not, was much more pronounced among the Russian patients (.95 vs 2.20, p = .000, F=30.60), than among the Norwegians (.47 vs .85, NS). Thus, the bonus of being employed appeared to be much greater for the Russian patients. The probable causes is that the more generous Norwegian social security arrangements make it possible for people without jobs to live an economically decent life, but with problems related to activities of daily living (HoNOS 10) as a consequence. Disability benefit recipiancy has increased in most OECD-countries despite improvement in most health indicators [47], and mental disorders account for up to one-third of the total disability pensions with depression is the major cause [48,49]. In Norway the figure is 29.7% and about 12 times more is spent on disability-related programs than on unemployment [47]. In Russia, invalidity due to psychiatric disorders is also increasing (with 36% from 1990 to 2000 [4]) and disabled status and disability pensions are also here to some extent used as survival strategies [50].

### Critical comments

The strength of the study was that (1) most of the acute wards in the relevant areas participated (Norway 4 out of 5, Russia 100%). (2) All committed patients took part in the study. (3) The clinicians who did the ratings were systematically trained in the use of the forms and instruments. (4) Written instructions, scorings criteria and support from the study group were easily available (local researchers, telephone). (5) In all the phases of the project, there was close contact between the Russian and the Norwegian study groups. Weaknesses were: (i) The differences in the training of professionals, and the organisation and capacity of the mental health services between Russia and Norway, may have effected the ratings of social and clinical problems [51]. (ii) Due to national adaptions to for example objective living conditions, some HoNOS-criterias may have been used differently. (iii) Although forward and back translations were used, linguistic misunderstandings may have occurred. (iv) Only clinical diagnoses were used, and Russian and Norwegians clinicians may use some diagnostic criteria differently [31]. (v) There may have been different thresholds between Russian and Norwegian patients for reporting mental problems [10].

## Competing interests

There are no competing interests in the study.

## Authors’ contribution

KWS led the data collection at one of the Norwegian hospitals, did the statistical analyses and wrote the draft of the manuscript. GR led the Russian part of the study, translated the MAP and the HoNOS into Russian, trained the Russian staff, and commented on the manuscript. AB led the data collection in the participating Russian wards, read and commented on the manuscript, TS contributed to the planning of the study and commented on the manuscript, TB led the datacollection at the other Norwegian hospital, read and commented upon the manuscript. All authors read and approved the final manuscript.
